# Conjugation frequency of ESBL- and pAmpC- *E. coli* in broiler chickens in vivo and in vitro

**DOI:** 10.1186/s12866-026-04822-1

**Published:** 2026-02-11

**Authors:** Diana Vargas, Roswitha Merle, Anika Friese, Uwe Roesler, Caroline Robé

**Affiliations:** 1 Institute of Animal Hygiene and Environmental Health, Veterinary Centre for Resistance Research, Freie Universität Berlin, Berlin, Germany; 2 Institute of Veterinary Epidemiology and Biostatistics, Veterinary Centre for Resistance Research, Freie Universität Berlin, Berlin, Germany

**Keywords:** Conjugation frequency, *Escherichia coli*, ESBL, pAmpC, *mcr-1*, Plasmid, Transconjugant, Antimicrobial resistance, Broiler chicken

## Abstract

**Background:**

Plasmid-mediated conjugation is a major form of horizontal gene transfer (HGT), facilitating dissemination of antimicrobial resistance (AMR) and the emergence of multi-drug-resistant (MDR) strains. In poultry, *Escherichia coli* producing extended-spectrum β-lactamases (ESBL) and plasmid-mediated AmpC β-lactamase (pAmpC) enzymes are common and contribute to antibiotic resistance. Additionally, plasmid-mediated colistin resistance gene *mcr-1* in poultry requires attention, as it is a last-resort antibiotic in human medicine. Although plasmid-mediated conjugation is known to play a role in spreading antimicrobial resistance, its specific impact on resistance transmission within the broiler microbiota is still not well understood. We assessed conjugation dynamics of the *mcr-1* gene from a pAmpC- *E. coli* to an ESBL- *E. coli* observed in an in vivo broiler chicken trial and compared them to conjugation frequencies under different in vitro conditions (LB broth, intestinal chicken cells in DMEM/F-12 medium, and DMEM/F-12 medium alone), with two initial bacterial loads.

**Results:**

From in vivo trial, among 138 broiler chickens sampled after a 49-day fattening period, transconjugants were detected in the cecal content of 35 broilers. The median conjugation frequency observed was of -5.02 log_10_ transconjugants/donor. Median conjugation frequencies across all in vitro conditions varied by less than one log unit (between − 6.8 and − 6.0 log_10_ transconjugants/donor), and no significant differences in conjugation efficiency were observed between initial bacterial concentrations.

**Conclusions:**

We confirmed bacterial conjugation between pAmpC-producing *E. coli* carrying the *mcr-1* gene and ESBL-producing *E. coli* both in vitro and in vivo. The similar conjugation efficiencies observed across different in vitro methods suggest that experimental conditions have minimal influence under controlled settings. In contrast, the in vivo results underscore the significance of the host’s physiological environment in HGT. The presence of transconjugants after a 49-day fattening period indicates that intestinal bacteria function as reservoirs for resistance plasmids and could facilitate their spread throughout the broiler production chain. However, limitations like the possibility of plasmid transfer to other bacteria, unknown persistence of the plasmid in the gut, and potential modulations of transfer efficiency under antibiotic selection must be considered when interpreting the results.

**Supplementary Information:**

The online version contains supplementary material available at 10.1186/s12866-026-04822-1.

## Introduction

Horizontal gene transfer (HGT) plays a critical role in bacterial evolution, facilitating the dissemination of antimicrobial resistance (AMR) [1]. Among the mechanisms of HGT, plasmid-mediated conjugation is particularly important due to its efficiency in transferring resistance genes across both intra- and interspecies bacterial populations [2]. This process poses a challenge to public health, as antibiotic-resistant bacteria, including multi-drug resistant (MDR), continue to emerge across human, animal, and environmental reservoirs [3]. Understanding the dynamics of plasmid transfer is therefore essential for guiding strategies to prevent the spread of AMR [4].

In livestock production, *Escherichia coli* harboring extended-spectrum β-lactamases (ESBL) and plasmid-mediated AmpC β-lactamase (pAmpC) genes have been detected and recognized as contributors to the spread of antibiotic resistance [5, 6]. ESBL genes are frequently associated with mobile genetic elements such as plasmids, facilitating horizontal transfer across bacterial populations [7, 8]. These determinants often encode resistance to antimicrobials, including third-generation cephalosporins [9]. According to the World Health Organization (WHO), these drugs are classified as ‘Highest Priority Critically Important Antimicrobials’. They are used as last-resort treatments for serious infections in humans, including those by MDR Gram-negative bacteria, and have few to no alternative therapies [10]. Broilers frequently harbor ESBL- and pAmpC- producing *E. coli* [11–13], with prevalence mainly driven by horizontal spread within broiler farms and vertical transmission from breeding stock [11, 14]. Resistant isolates have been detected not only in feces, litter, and farm equipment, but also in the surroundings of broiler farms [15–17]. Moreover, the plasmid-mediated colistin-resistant gene *mcr-1* has been reported in broiler production, primarily associated with veterinary use of the drug, which selects for strains harboring plasmid-mediated *mcr* genes [18, 19]. Importantly, recent surveillance data from Germany indicate a declining trend in *mcr-1* prevalence [20]. Given that colistin serves as a last-resort antibiotic in human medicine, its occurrence in broilers poses a public health concern, making the monitoring of its occurrence essential [21]. ESBL, pAmpC, and *mcr* genes represent a potential route of zoonotic transmission to humans through direct contact or contaminated meat [22–25]. However, despite the recognized role of plasmid-mediated conjugation in resistance dissemination, the extent to which this mechanism contributes to the spread of antimicrobial resistance within the chicken cecal microbiota remains insufficiently characterized [26, 27].

Experimental studies on plasmid conjugation are typically performed under in vitro conditions using standardized laboratory media and controlled environments [28]. While such approaches are useful for mechanistic investigations, they may not accurately reflect the complex ecological and physiological factors present in the gastrointestinal tract of poultry or other animals. In vivo studies offer a more realistic perspective but are less commonly conducted due to logistical and ethical constraints [29, 30]. Moreover, in both in vivo and in vitro settings, distinguishing whether a resistant isolate emerged from a recent HGT event or originated from clonal expansion of an ancestral strain that had previously acquired the resistance gene remains highly challenging [31, 32].

The dissemination of AMR through horizontal gene transfer in broilers remains insufficiently characterized, particularly regarding the recovery of transconjugant isolates following experimental inoculation and throughout the fattening period. In this study, we aimed to estimate the frequency of *mcr-1* gene transfer between a donor pAmpC- and recipient ESBL-producing *E. coli* originating from broiler chickens under controlled in vitro conditions and to compare these results with observations obtained from an in vivo trial. This comparison provides insight into how efficiently antimicrobial resistance genes may disseminate within the broiler and to which extent laboratory-derived estimates reflect in vivo dynamics [26, 33].

## Methods

### Bacterial strains

Experiments both in vivo and in vitro were conducted using two *E. coli* strains previously isolated from healthy broiler chickens during earlier research projects [34, 35]. An ESBL-producing *E. coli* belonging to sequence type (ST) 410 and phylogenetic group A, served as the recipient strain harboring the chromosomally encoded *bla*_CTX−M−15_ gene, which confers resistance to cephalosporins; the strain also demonstrated phenotypic resistance to enrofloxacin [34, 36]. A pAmpC-producing *E. coli* of ST10 and phylogenetic group A, used as a donor strain carrying resistance genes *mcr-1*, which mediates resistance to colistin, and *bla*_*CMY−2*_, and *bla*_*TEM−1*_, encoding resistance to cephalosporins [36]. For selective recovery during the experiments, enrofloxacin was used to isolate the ESBL-producing *E. coli* strain, allowing its growth while preventing growth of the pAmpC-producing *E. coli* strain. Conversely, colistin was used to recover the pAmpC-producing donor strain, permitting its growth while inhibiting the ESBL- producing strain.

### In vivo bacterial conjugation

The in vivo transconjugants analyzed in the present study derived from a previously conducted in vivo trial. All experimental procedures were authorized by the German Animal Ethics Committee of the Regional Office for Health and Social Affairs Berlin (“Landesamt für Gesundheit und Soziales”, LAGeSo; approval number G0079/23). All animal handling and housing followed the national regulations and institutional guidelines of Freie Universität Berlin regarding animal welfare. The trial evaluated the efficacy of a single-strain live *E. coli* vaccine as a competitive exclusion (CE) strategy to reduce intestinal colonization by ESBL- and pAmpC-producing *E. coli* in broiler chickens using the two strains mentioned above. Ranger Gold broiler chicken eggs were obtained from a commercial hatchery in Germany for the animal experiment [37]. 

Briefly, prior to starting the trial, all rooms, equipment, and materials were confirmed free of ESBL- and pAmpC-producing bacteria, as previously described in Vargas et al. [37]. ESBL- and pAmpC-negative Ranger Gold broiler chickens were assigned to two experimental groups and a positive control group (46 birds per group). One experimental group received the vaccine via coarse spray on day one of life, while the other experimental group was administered the vaccine through drinking water on day five of life. The positive control remained unvaccinated. On day three, all groups were orally co-inoculated with an equal mixture of the two ESBL- and pAmpC-producing *E. coli* strains at a dose of 10² CFU per bird, with each bird receiving 200 µL of the bacterial suspension via a crop needle. The inoculum was prepared following the protocol described by Robé et al. [38].

To assess colonization by ESBL-/pAmpC-producing *E. coli*, cloacal swabs were obtained from all broilers starting on day 4 of life. Daily cloacal swab sampling was conducted through day 8, after which samples were collected at weekly intervals on days 9, 16, 23, 30, and 37 of life. After a 49-day fattening period, necropsies were performed of all broilers, and luminal contents of the cecum were collected to enumerate ESBL- and pAmpC-producing *E. coli* in all groups. The plate set used included a triple antibiotic chromogenic agar plate (CHROMagar Orientation, Mast Diagnostica, Reinfel, Germany) supplemented with a combination of three antibiotics (2 µg/mL cefotaxime (Thermo Scientific, Waltham, MA, USA), 7 µg/mL colistin (Carl Roth GmbH, Karlsruhe, Germany), and 4 µg/mL enrofloxacin (Sigma-Aldrich, St. Louis, MO, USA)). Indeed, no growth of the ESBL-E. *coli* recipient strain (susceptible to colistin; resistant to cefotaxime and enrofloxacin) or the pAmpC-*E. coli* donor strain (susceptible to enrofloxacin; resistant to cefotaxime and colistin) was expected, while transconjugants were resistant to the three antibiotics due to the transfer of the *mcr-1* plasmid (encoding resistance to colistin) from the pAmpC-*E. coli* donor strain to the ESBL-*E. coli* recipient strain. Each colony from these plates was re-streaked onto a fresh chromogenic agar plate containing the triple combination of antibiotics and subsequently cultured in Luria-Bertani broth (LB) (Carl Roth, Karlsruhe, Germany) supplemented with the same antibiotics before being stored at − 80 °C for further confirmation of transconjugant analyses. Antibiotic concentrations were selected to be low enough to allow growth of all resistant bacteria, yet high enough to inhibit susceptible strains, minimizing interference from other bacteria of the microbiota.

### In vitro bacterial conjugation

#### Bacterial inoculum preparation

Both ESBL-/pAmpC*-E. coli* strains were streaked individually from stock cultures onto plain Columbia agar plates with 5% sheep blood (Oxoid, Wesel, Germany) containing no antibiotics and incubated at 37 °C for 18–24 h. One colony from each strain was subsequently used to inoculate 5 mL of LB broth, followed by overnight incubation under shaking conditions (150 rpm) at 37 °C. Overnight cultures were diluted in fresh LB broth to an initial optical density at 600 nm (OD_600_) of 0.03–0.04 and incubated with shaking until reaching an OD_600_ of 0.6, corresponding to approximately 2 × 10⁸ CFU/mL. Each strain was independently quantified using 10-fold serial dilutions and plated on LB agar supplemented with antibiotics to ensure bacterial concentrations. The ESBL- *E. coli* recipient strain was plated on LB agar supplemented with 4 µg/mL enrofloxacin, while the pAmpC*-E. coli* donor strain was cultured on LB agar containing 2 µg/mL colistin. One milliliter of each culture was harvested by centrifugation at 7000 × g for 10 min at 4 °C and washed in phosphate-buffered saline (PBS; Oxoid, Wesel, Germany). The washed cells were then resuspended in either LB or DMEM/F-12 medium and used as inoculum for bacterial conjugation assays. Transconjugants were selected on LB agar supplemented with enrofloxacin (4 µg/mL) and colistin (2 µg/mL). All experiments were carried out using seven independent biological replicates, each accompanied by three technical replicates.

#### Bacterial conjugation in liquid medium

To assess the impact of initial bacterial concentration on transfer frequencies, two bacterial loads were tested. Washed cells from each strain were first resuspended in 1 mL of LB broth. To achieve a final initial concentration of 10⁵ CFU/mL, 10 µL of each resuspended strain was added to 9980 µL of fresh LB broth. For a concentration of 10⁶ CFU/mL, 100 µL of each strain was mixed with 9800 µL of LB broth. The resulting suspensions were incubated at 41 °C with shaking at 150 rpm for 4 h. The temperature of 41 °C was selected to mimic the internal body temperature of broiler chickens. Following incubation, appropriate serial dilutions were plated on LB agar supplemented with selective antibiotics and incubated at 37 °C to enumerate donor and recipient populations, as well as transconjugants.

#### Bacterial conjugation in intestinal chicken cells and cell-free media

Intestinal chicken cells CHIC Clone-8E11 ((CHIC) Tentamus Pharma & Med Deutschland GmbH) were grown in T75 cell culture flasks (Sarstedt, Nümbrecht, Germany) and maintained in DMEM/F-12 medium (Dulbecco’s Modified Eagle Medium/Nutrient Mixture F-12, Gibco, Thermo Fisher Scientific, Waltham, MA, USA) supplemented with 20% fetal bovine serum. For conjugation assay, cells were seeded in 12-well cell culture plates (standard surface, flat base; Sarstedt, Nümbrecht, Germany) at a density sufficient to reach 75% confluence. Prior to bacterial inoculation, cells were washed twice with PBS.

Using the bacterial inoculum describe in the section *“Bacterial inoculum preparation”*, bacterial suspensions were standardized and combined in equal volumes to achieve multiplicities of infection (MOI) of 1 and 10, corresponding to final concentrations of 10⁵ and 10⁶ CFU/mL, respectively. Washed cells were resuspended in 1 mL of fresh DMEM/F12 medium. For the MOI 1, 5 µL of each strain was diluted in 990 µL of DMEM/F12 medium, while for the MOI 10, 50 µL of each strain was mixed with 900 µL of DMEM/F12 medium. In both cases, 1 mL of the prepared bacterial suspensions was added to wells containing CHIC or DMEM/F-12 medium alone. Control wells included CHIC only (no bacteria added). Plates were incubated at 37 °C with 5% CO₂ under standard culture conditions, following the manufacturer’s instructions, for 4 h. After incubation, supernatants were collected from each well, serially diluted, and plated on selective LB agar to quantify donor, recipient, and transconjugant populations, as described above.

### Confirmation of transconjugant isolates from in vivo and in vitro conjugation assays

To verify the identity of resultant transconjugants, each colony was individually inoculated into 200 µL of LB containing both antibiotics (enrofloxacin 4 µg/mL and colistin 2 µg/mL) in 96-well plates (standard surface, round base; Sarstedt, Nümbrecht, Germany), including appropriate negative and positive controls. Plates were incubated overnight without shaking at 37 °C. The following day, wells were visually inspected for bacterial growth by comparing turbidity and pellet formation to positive and negative controls. To confirm the ESBL recipient strain, a multiplex real time PCR targeting the *bla*_CTX-M_, *bla*_TEM_, and *bla*_CMY_ genes was performed according to the protocol described by Roschanski et al. [39]. In addition, conventional PCR was conducted to detect the presence of the *mcr-1* plasmid, following the method outlined by Rebelo et al. [40]. Additionally, for transconjugants recovered from the in vivo trial, colony morphology and color on chromogenic agar were compared to the inoculated strains, and phenotypic growth on plates containing cefotaxime, enrofloxacin, and colistin was assessed. Because the ESBL recipient strain carries chromosomally encoded resistance to cephalosporins and fluoroquinolones, these phenotypic observations further supported that the identified transconjugants were derived from the recipient strain. Furthermore, we verified that no other ESBL- or pAmpC-producing strains were present in the broilers or experimental facilities prior to starting the trial.

### Analysis of conjugation frequency

Bacterial conjugation frequencies were determined by calculating the number of transconjugants divided by the total number of donor cells at the end of the experiment [41–43]. According to Kosterlitz et al. [44], this represents a conjugation frequency expressed as a population ratio, as it reflects the relative success of plasmid transfer into a new host at a single time point. This approach allowed to evaluate plasmid transfer at defined endpoints: at the end of the fattening period in vivo and after four hours of incubation in the in vitro assays.

### Statistical analysis

The log₁₀-transformed conjugation frequency values were used for all statistical analyses. After confirming that the data met the assumption of normal distribution, a one-way ANOVA was performed to evaluate differences in conjugation frequencies across the six in vitro experimental conditions. These included co-incubation with CHIC, incubation in DMEM/F-12 medium (without CHIC), and incubation in LB broth, each tested at two initial bacterial concentrations (10⁵ and 10⁶ CFU/mL). Post hoc pairwise comparisons were conducted using the Hochberg correction to account for multiple testing. Statistical significance was set at *p* < 0.05.

All statistical analyses were performed using IBM SPSS Statistics software, version 25.0 (SPSS, Inc., Chicago, IL, USA). Graphical representations were generated using R software (R Core Team, 2023, Vienna, Austria).

## Results

### Conjugation frequency in vivo

**Fig. 1:**
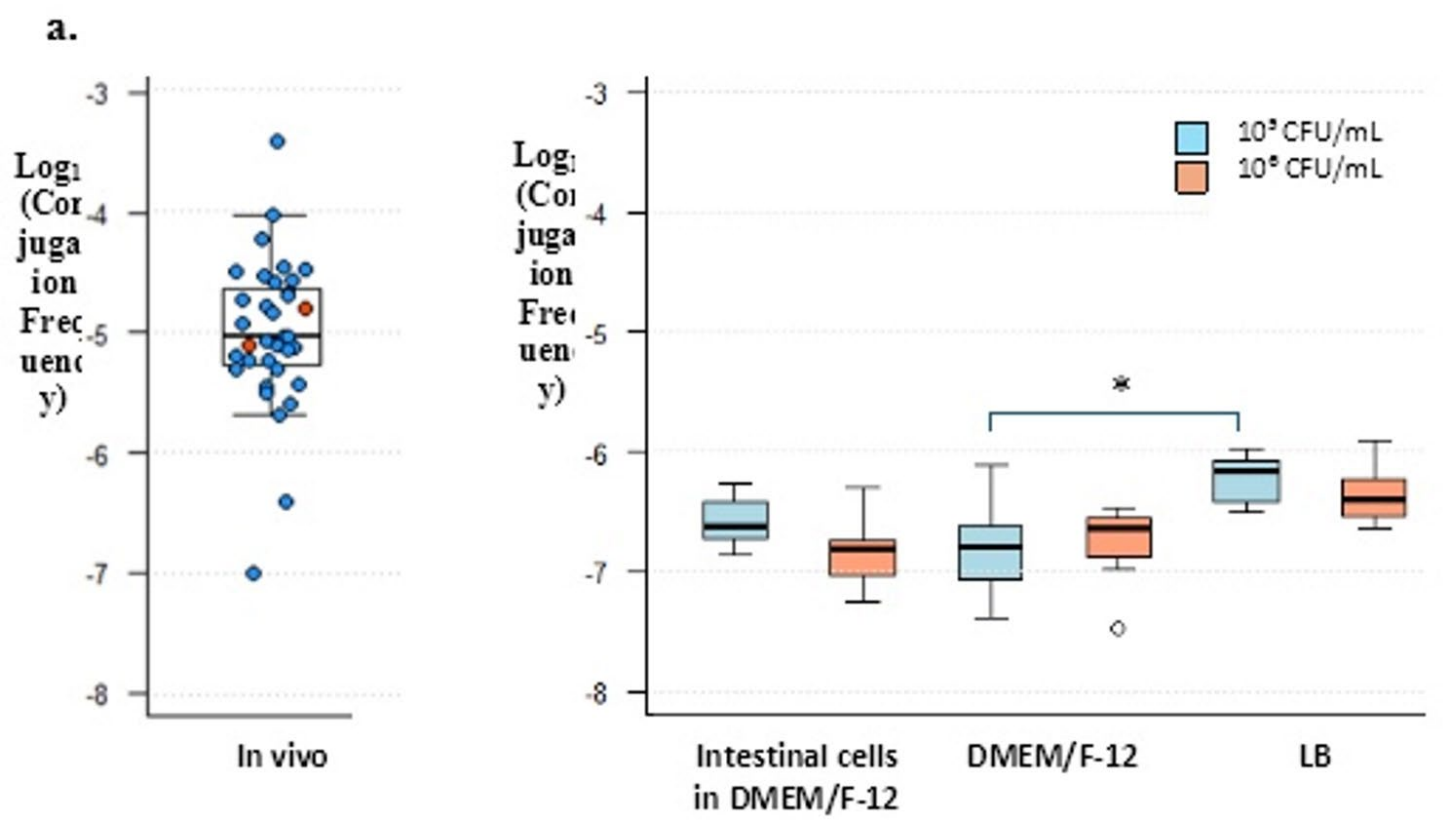
a. Conjugation frequency (Log_10_ transconjugants/donor) of the *mcr-1* gene from donor pAmpC- producing *E. coli* to recipient ESBL- producing *E. coli* in the cecum following a 49-day fattening period in vivo. Box plot shows the distribution of values, with individual data points indicated as colored dots: blue represents 33 transconjugant-positive broilers from the group that received the CE via drinking water on day five of life, and red represents 2 broilers from group administered the CE product via coarse spray on day one of life. **b**. Conjugation frequency under in vitro conditions including intestinal chicken cells in DMEM/F-12 medium, DMEM/F-12 medium alone, and LB broth using two initial bacterial concentrations: 10⁵ CFU/mL (blue) and 10⁶ CFU/mL (orange). Boxes illustrate the interquartile range, with the median conjugation frequency marked by a horizontal line within each box. Whiskers span from the 5th to the 95th percentile, while data points outside this range are displayed individually as outliers. * *p =* 0.02 (Hochberg-adjusted)

 A total of 78 isolates were ultimately confirmed as true transconjugants. Among these, 68 were recovered from cecal samples of 35 broilers, while 10 originated from cloacal swabs of 7 broilers. The presence of transconjugants in the cecal contents of these 35 animals, reflected the transfer of the *mcr-1* gene from the donor pAmpC- to the recipient ESBL- producing *E. coli* after the fattening period of 49 days. Two animals belonged to the experimental group that received the CE via spray on day 1, while the remaining 33 were from the group that received the CE via drinking water on day 5. No transconjugants were detected in the cecal content of the positive control group untreated with the CE (S. Table 1). The calculated conjugation frequencies for these 35 broilers ranged from a minimum of −7.00 log_10_ to a maximum of −3.43 log_10_, yielding a total range of 3.57 log_10_ units across the 35 broilers. The median conjugation frequency was − 5.02 log_10_ transconjugants/donor. The interquartile range, representing the middle 50% of the log₁₀-transformed values, extended from − 5.31 to − 4.59 log_10_ (Fig. 1. a., S. Table 3).

Transconjugants detected in cloacal swabs from seven animals had the first isolate recovered on day 9 of life (corresponding to day 6 post inoculation with ESBL-/pAmpC-*E. coli*). Six of these animals belonged to the CE drinking water group, five of which were also positive in cecal contents, while one animal was positive only in the cloacal swab. Furthermore, cloacal swab positivity was detected in a single animal from the positive control group. In addition, multiplex PCR revealed that some transconjugant isolates carried not only the *mcr-1* gene but also further resistance determinants originating from the pAmpC-*E. coli* donor, including *bla*_TEM_ and *bla*_CMY_. From cecal samples, 13 isolates tested positive for both *bla*_TEM_ and *bla*_CMY,_four isolates were additionally positive only for *bla*_CMY,_and 14 isolates were positive for *bla*_TEM_. From cloacal swabs, three isolates additionally tested positive for *bla*_CMY_ (S. Table 1).

### Conjugation frequency in vitro

The efficiency of *mcr-1* gene transfer from the pAmpC-*E. coli* donor to the ESBL-*E. coli* recipient strain was assessed under three experimental conditions: in the presence of CHIC with DMEM/F-12 medium, in the absence of CHIC using only DMEM/F-12 medium, and in LB broth alone. Additionally, two initial bacterial concentrations (10⁵ and 10⁶ CFU/mL) were tested under each of these conditions to evaluate the influence of the starting inoculum on conjugation frequency. 

Conjugation frequency for *mcr-1* in LB broth yielded median values of −6.16 and − 6.41 log_10_ transconjugants/donor for initial bacterial concentrations of 10⁵ and 10^6^ CFU/mL, respectively, with no statistical difference observed between the tested initial bacterial concentrations (Hochberg-adjusted *p* > 0.999) (Fig. 1. b., Table S4, Table S5, and Table S6). Median values for CHIC were − 6.63 and − 6.81 log_10_ transconjugants/donor, and for DMEM/F-12 medium, −6.80 and − 6.65 log_10_ transconjugants/donor at initial bacterial concentrations of 10⁵ and 10^6^ CFU/mL, respectively (Fig. 1. b.and S. Table 4). The median conjugation frequencies among all tested conditions remained within one log unit, with no statistically significant variation observed, except for a difference between DMEM/F-12 medium (10⁵ CFU/mL) and LB broth (10⁵ CFU/mL) (Hochberg-adjusted: *p =* 0.02) (Fig. 1. b. , Table S4, Table S5, and Table S6).

 The transfer of *bla*_*TEM*_ and *bla*_*CMY*_ was also observed in some isolates. From the CHIC conjugation assay, four isolates carried additionally both *bla*_*TEM*_ and *bla*_*CMY*_, while 15 isolates harbored *bla*_*TEM*_. Six isolates from the conjugation assay conducted in DMEM/F-12 medium were also positive for *bla*_*TEM*_ and *bla*_*CMY*_, and 19 isolates were positive for *bla*_*TEM*_ (S. Table 2).

## Discussion

We investigated the conjugation frequencies of *mcr-1* transfer from donor pAmpC- to a recipient ESBL- producing *E. coli* under different in vitro conditions, following the detection of transconjugants in an in vivo broiler trial. This approach enabled us to confirm the transferability of the *mcr-1*-carrying plasmid, quantify conjugation frequencies, and assess the impact of varying experimental conditions on plasmid-mediated gene transfer.

We recovered transconjugants from the cecal content of broilers in our in vivo trial and from three different experimental conditions in vitro. In both cases, isolates likely originated either from recent HGT events or from clonal expansion of an ancestor that had previously acquired the plasmid. Conjugation frequencies of approximately − 5 log₁₀ CFU/mL were detected in vivo. Although such frequencies may appear low, even infrequent conjugation events can have significant implications in the context of poultry farming [45]. Conjugation frequencies from in vivo likely result from factors absent in vitro, such as dense mucosal microenvironments, prolonged contact between donor and recipient cells, metabolic activity, antibiotics, biofilms, and temperature [46]. Following plasmid acquisition, bacteria can lose it during cell division or in the presence of selective pressures, particularly if maintaining the plasmid imposes a fitness cost [47, 48]. Additionally, colonization within the host can be transient, with plasmid-carrying bacteria fluctuating over time depending on bacterial competition, nutrient availability, or host immune response [49, 50]. Consequently, the observed frequency may be lower due to clonal expansion of a few transconjugants, or higher initially but declines as transconjugants lose the plasmid or no longer require it for survival. Conjugation frequencies in vitro included: standard LB broth, CHIC with DMEM/F-12 medium, and DMEM/F-12 medium alone. Across all conditions, conjugation frequencies remained consistently low, with no significant difference observed between all the models. A statistical difference was detected between DMEM/F-12 medium (initial bacterial concentration of 10⁵ CFU/mL) and LB broth (also 10⁵ CFU/mL); however, the median values differed by less than one log unit, suggesting a minor difference in the overall conjugation frequencies [51]. These findings possibly imply that factors beyond nutrient availability and physical proximity between cells may constrain plasmid transfer under in vitro conditions. Although LB broth provides abundant nutrients, it lacks complex physiological signals characteristic of the intestinal environment [29]. Similarly, while epithelial cell cultures offer greater biological relevance, they still fail to replicate the full range of interactions between the host and the gut microbiota [29]. In our in vitro conjugation experiments using intestinal epithelial cell lines, only the supernatant was collected and analyzed for the presence of transconjugants. Bacteria that may have adhered to the cell monolayer or engaged in localized conjugation events could have been missed in the analysis. Similarly, in the in vivo trial, only the luminal content of the cecum was collected, without disturbing or scraping the mucosal layer of the cecum. Consequently, both approaches may underestimate the actual occurrence of plasmid-mediated gene transfer. Moreover, it is important to note that the conjugation assay on CHIC was conducted at 37 °C following the manufacturer’s recommendations to maintain optimal cell viability, rather than at 41 °C to mimic broiler body temperature. This limitation indicates that the in vitro conditions do not fully replicate the in vivo environment, where higher temperatures and additional host factors may influence bacterial behavior, including conjugation efficiency.

A total of 1,947 colonies were initially recovered on the triple-antibiotic selective plates from cloacal swab and cecal samples; however, only 78 were ultimately confirmed as true transconjugants. The high proportion of false positives is likely attributable to the nature of the original samples, which contained substantial organic material capable of reducing the effective antibiotic concentration in the agar, thereby allowing non-transconjugant colonies to grow. In addition, initial plating of undiluted or minimally diluted samples to maximize transconjugant recovery may have further facilitated the growth of non-target bacteria. In the in vivo study, cloacal swabs were collected regularly from all broilers throughout the trial period. The swabs revealed the presence of transconjugants in a total of seven broilers, with the first one detected on day 9 of life, and most cases observed in the second half of the fattening period (days 23 to 37 of life). These findings align with those of Hadziabdic et al. [52], who also detected transconjugants at an early stage, but observed the majority toward the end of the trial, while investigating the in vivo transfer of avian native IncA/C_2_ *bla*_NDM−1_- carrying plasmid pRH-1238. However, cloacal swabs usually collect a limited and inconsistent sample, primarily from the distal gut and cloacal surface, which may not represent total cecal bacterial loads [53–55]. Therefore, quantification is generally unreliable, and in our study, swabs only indicated the presence of transconjugants rather than quantifying them. Cecal sampling allows for a more accurate and quantitative assessment of bacterial load [54, 56]. When analyzing cecum samples, plasmid transfer was detected in 35 out of 138 broilers on day 49, which aligns with the low detection rates also observed by Netherwood et al. [57], who investigated the potential for gene transfer from the genetically modified probiotic *Enterococcus faecium* (NCIMB 11508) to other gut bacteria, reporting positive transfer in only 5 out of 72 chickens. In our trial, transconjugants were detected in cecal samples from 33 broilers in the CE drinking water group, compared with only two in the coarse spray group, while none were identified in the positive control group. As previously shown, at the end of the animal trial, cecal colonization by both the ESBL- and pAmpC-producing *E. coli* strains differed among experimental groups, with the highest levels observed in the drinking water group, followed by the coarse spray group, and substantially lower levels in the positive control group. Moreover, following inoculation, the ESBL-producing recipient strain established more rapidly in the CE drinking water group, whereas establishment was delayed in the other groups. These differences in colonization dynamics may have contributed to the observed variation in transconjugant detection at the end of the fattening period, potentially due to levels remaining below our detection limit. The detection limits were 10 CFU/mL for the in vitro assays and 20 CFU/g for cecal samples [37].

In the in vivo setting, transconjugants were recovered after 49 days, whereas in vitro isolates were obtained after only 4 h. These markedly different timeframes allow for distinct biological processes to influence plasmid persistence and detectability. In vivo, bacteria may have had a prolonged period during which plasmid acquisition, loss, or stabilization could occur, alongside potential clonal expansion or displacement by competing strains [29, 58]. For liquid conjugation assays, it is recommended to keep short time incubations to avoid nutrient depletion and ensure a well-mixed culture [59]. Time consideration is mainly important when using cell culture models, as longer incubations can compromise cell viability. Under in vitro conditions, a rapid initial phase of plasmid transfer followed by a plateau in transconjugant numbers has been observed. Headd et al. [60] demonstrated that transconjugant formation between *E. coli* strains carrying IncFII plasmids increases over the first few hours but reaches a steady state between 4 and 8 h of incubation, indicating that further donor–recipient contact beyond this period does not significantly enhance plasmid transfer. Low conjugation frequencies across the three in vitro conditions were observed. Frequencies ranged approximately from 10⁻⁷ to 10⁻⁶ transconjugants/donor, which is comparable to the findings reported by Saliu et al. [43]. After 4 h of in vitro co-incubation between ESBL- and AmpC-producing *E. coli* donors and various *Enterobacteriaceae* recipients, frequencies ranged roughly from 10⁻⁵ to 10⁻⁶ per donor, depending on the specific strain pair. This temporal limitation of in vitro conjugation may partly explain why such experiments often fail to fully replicate the complex dynamics of plasmid transmission observed in vivo, particularly in the avian gut, where broilers with extended fattening periods may provide different HGT dynamics that are not captured within short incubation assays. In general, the gastrointestinal tract constitutes a dynamic and complex ecosystem, with secretion of enzymes and biofilm formation, all of which could create a favorable environment for gene transfer [30, 46, 61].

The selective antibiotic combination used in agar plates for processing the in vivo samples enabled the detection of the *mcr-1* plasmid, allowing us to specifically assess the transfer of this resistance gene. However, ESBL-producing *E. coli* strains associated with poultry are well-documented to harbor one or multiple plasmids simultaneously [62]. Our donor pAmpC- producing *E. coli* strain was known to harbor additional resistance determinants, including *bla*_CMY−2_and *bla*_TEM-1_. During PCR multiplex screening, some transconjugant isolates from the in vivo and in vitro experiments were found to carry not only the *mcr-1* gene but also these additional resistances, indicating the co-transfer of multiple determinants. Schaufler et al. [63] demonstrated that ESBL-producing *E. coli* can carry antibiotic resistance on several plasmids without any measurable fitness cost under laboratory conditions. Likewise, Benz et al. [64] reported that transconjugants carrying ESBL-plasmids did not show evidence of a fitness cost. Moreover, in specific lineages, the presence of ESBL plasmids was associated with enhanced virulence traits, such as increased biofilm formation, improved survival under host-like conditions, and greater pathogenic potential in infection models [63, 65]. It is also important to note that, in the in vivo trial, transconjugants were detected following the use of two defined *E. coli* strains. However, conjugative events may have occurred in other *E. coli* and bacterial populations that went undetected. This includes isolates unable to grow under our plating conditions, as well as populations that were in a low metabolic activity or cellular conditions unfavorable for plasmid exchange at the time of sampling [60]. This highlights the possibility that the actual frequency and impact of gene transfer in complex microbial ecosystems may be underestimated by targeted assays.

In the in vivo study, broilers were inoculated with an equal mixture of both bacterial strains at a concentration of 10² CFUs. A very low bacterial dose was used because it has been previously shown to reliably establish colonization in chickens, while also reflecting realistic farm conditions. It was demonstrated that, despite this low inoculation, bacterial counts in the cecum increased substantially, reaching up to 10⁹ CFU/g, indicating extensive in vivo replication of *E. coli* in the gut [38]. Higher bacterial concentrations like those used in vitro, might have increased conjugation frequencies; however, using such doses in vivo would not reflect natural exposure on farms and could result in unnaturally high colonization levels. To assess the influence of bacterial cell density on plasmid transfer, we conducted in vitro conjugation assays using two initial concentrations: 10⁵ and 10⁶ CFU/mL. These relatively low densities were chosen based on the study by Saliu et al. [43], who used similar concentrations, to mimic realistic infection scenarios in poultry. *E. coli* has been reported at densities ranging from 10⁶ to 10⁸ cells per milliliter in the cecum of chickens [66]. Notably, we observed no significant difference in conjugation efficiency between these two conditions, suggesting that within this density range, bacterial abundance was not a limiting factor for plasmid transfer. This observation aligns with recent findings from Rodriguez-Grande et al. [67], who investigated how physical interaction dynamics between bacterial cells affect the efficiency of conjugative plasmid transfer. They imply that plasmid conjugation is density-dependent primarily at very low cell concentrations; once a certain threshold is reached , further increases in donor and recipient density do not result in proportionally more transconjugants.

Important limitations should be considered when interpreting our results. In this study, transconjugant isolates were re-streaked on agar plates containing a combination of three antibiotics, cefotaxime, colistin, and enrofloxacin. However, no phenotypic susceptibility testing (MIC assays) was performed, meaning that the presence of resistance genes indicates potential rather than confirmed functional resistance. Additional limitations include the possibility of plasmid transfer to other *E. coli* strains or different bacterial species, the duration of plasmid persistence in the gut, and how plasmid transfer dynamics may be influenced under antibiotic selective pressure. Finally, only the luminal content of the cecum was collected, without sampling or scraping the mucosal layer, where transconjugants may have been present.

 In conclusion, we demonstrated bacterial conjugation between a pAmpC-*E. coli* donor strain carrying the *mcr-1* gene and ESBL-*E. coli* recipient strain both in vivo and in vitro. The lack of significant differences among the in vitro methods suggests that experimental conditions have a limited impact on conjugation efficiency under controlled settings. Observed frequencies in vivo highlight the importance of accounting for the physiological complexity of the host environment when assessing horizontal gene transfer. The detection of transconjugants after a 49-day fattening period indicates that broilers can act as reservoirs for resistance genes, which may pose a risk to public health. 

## Supplementary Information

Supplementary Material 1.

## Data Availability

All information produced or examined in this research is provided within the article and its supplementary documents. Additional data can be requested from the corresponding author on reasonable request.
