# Supplementary material for: Conjugation frequency of ESBL- and pAmpC- E. coli in broiler chickens in vivo and in vitro
Source: BMC Microbiol. 2026 Feb 11;26:293. doi: 10.1186/s12866-026-04822-1 (PMC13041292; doi:10.1186/s12866-026-04822-1)
Supplement: Supplementary file 1 — Supplementary Material 1. [file 12866_2026_4822_MOESM1_ESM.docx]

**Supplementary data**

**S. Table 1.** Resistance genes detected in 35 broilers after a fattening period of 49 days.

| **Animal number^*^** | **Transconjugant**  **ID** | **Origin of sample** | **Resistance genes detected^+^** |
| --- | --- | --- | --- |
| **414** | 1 | Cecum | *bla_CTX-M_, mcr-1* |
|  | 2 | Cecum | *bla_CTX-M_, mcr-1* |
| **433** | 1 | Cecum | *bla_CTX-M_, mcr-1* |
|  | 2 | Cecum | *bla_CTX-M_, mcr-1* |
| **502** | 1 | Cecum | *bla_CTX-M_, mcr-1, bla_TEM,_ bla_CMY_* |
| **503** | 1 | Cecum | *bla_CTX-M_, mcr-1, bla_TEM,_ bla_CMY_* |
| **505** | 1 | Cecum | *bla_CTX-M_, mcr-1, bla_TEM,_ bla_CMY_* |
|  | 2 | Cecum | *bla_CTX-M_, mcr-1* |
|  | 3 | Cecum | *bla_CTX-M_, mcr-1, bla_TEM,_ bla_CMY_* |
| **506** | 1 | Cecum | *bla_CTX-M_, mcr-1, bla_TEM,_ bla_CMY_* |
|  | 2 | Cloacal swab on day 30 | *bla_CTX-M_, mcr-1* |
| **507** | 1 | Cecum | *bla_CTX-M_, mcr-1, bla_TEM_* |
|  | 2 | Cecum | *bla_CTX-M_, mcr-1, bla_TEM_* |
|  | 3 | Cecum | *bla_CTX-M_, mcr-1, bla_TEM_* |
|  | 4 | Cecum | *bla_CTX-M_, mcr-1, bla_TEM_* |
| **508** | 1 | Cecum | *bla_CTX-M_, mcr-1, bla_TEM_* |
|  | 2 | Cloacal swab on day 30 | *bla_CTX-M,_  mcr-1* |
| **509** | 1 | Cecum | *bla_CTX-M_, mcr-1, bla_CMY_* |
|  | 2 | Cecum | *bla_CTX-M_, mcr-1, bla_CMY_* |
|  | 3 | Cecum | *bla_CTX-M_, mcr-1, bla_TEM,_  bla_CMY_* |
|  | 4 | Cecum | *bla_CTX-M_, mcr-1, bla_CMY_* |
|  | 5 | Cloacal swab on day 16 | *bla_CTX-M_, mcr-1* |
| **511** | 1 | Cecum | *bla_CTX-M_, mcr-1* |
| **512** | 1 | Cecum | *bla_CTX-M_, mcr-1* |
|  | 2 | Cecum | *bla_CTX-M_, mcr-1* |
|  | 3 | Cecum | *bla_CTX-M_, mcr-1* |
| **513** | 1 | Cecum | *bla_CTX-M_, mcr-1* |
| **516** | 1 | Cecum | *bla_CTX-M_, mcr-1* |
|  | 2 | Cecum | *bla_CTX-M_, mcr-1* |
|  | 3 | Cecum | *bla_CTX-M_, mcr-1* |
|  | 4 | Cecum | *bla_CTX-M_, mcr-1* |
|  | 5 | Cecum | *bla_CTX-M_, mcr-1* |
| **517** | 1 | Cecum | *bla_CTX-M_, mcr-1, bla_TEM,_ bla_CMY_* |
|  | 2 | Cecum | *bla_CTX-M_, mcr-1, bla_TEM,_ bla_CMY_* |
| **519** | 1 | Cecum | *bla_CTX-M_, mcr-1, bla_TEM_* |
| **521** | 1 | Cecum | *bla_CTX-M_, mcr-1* |
| **522** | 1 | Cecum | *bla_CTX-M_, mcr-1* |
|  | 2 | Cloacal swab on day 9 | *bla_CTX-M_, mcr-1* |
| **523** | 1 | Cecum | *bla_CTX-M_, mcr-1* |
| **524** | 1 | Cecum | *bla_CTX-M_ mcr-1, bla_CMY_* |
| **525** | 1 | Cecum | *bla_CTX-M_, mcr-1, bla_TEM_* |
|  | 2 | Cecum | *bla_CTX-M_, mcr-1, bla_TEM,_ bla_CMY_* |
|  | 3 | Cecum | *bla_CTX-M_, mcr-1, bla_TEM,_ bla_CMY_* |
|  | 4 | Cecum | *bla_CTX-M_, mcr-1, bla_TEM,_ bla_CMY_* |
| **526** | 1 | Cecum | *bla_CTX-M_, mcr-1* |
|  | 2 | Cecum | *bla_CTX-M_, mcr-1* |
| **527** | 1 | Cecum | *bla_CTX-M_, mcr-1* |
| **528** | 1 | Cecum | *bla_CTX-M_, mcr-1* |
| **529** | 1 | Cecum | *bla_CTX-M_, mcr-1, bla_TEM_* |
|  | 2 | Cecum | *bla_CTX-M_, mcr-1, bla_TEM_* |
|  | 3 | Cecum | *bla_CTX-M_, mcr-1, bla_TEM_* |
|  | 4 | Cecum | *bla_CTX-M_, mcr-1, bla_TEM_* |
|  | 5 | Cloacal swab on day 37 | *bla_CTX-M_, mcr-1, bla_CMY_* |
| **530** | 1 | Cecum | *bla_CTX-M_, , mcr-1, bla_TEM_* |
| **531** | 1 | Cecum | *bla_CTX-M_, mcr-1* |
|  | 2 | Cecum | *bla_CTX-M_, mcr-1* |
|  | 3 | Cecum | *bla_CTX-M_, mcr-1* |
| **532** | 1 | Cloacal swab on day 37 | *bla_CTX-M_, mcr-1* |
|  | 2 | Cloacal swab on day 37 | *bla_CTX-M,_  mcr-1* |
|  | 3 | Cloacal swab on day 37 | *bla_CTX-M_, mcr-1* |
| **534** | 1 | Cecum | *bla_CTX-M_, mcr-1* |
|  | 2 | Cecum | *bla_CTX-M_, mcr-1* |
| **536** | 1 | Cecum | *bla_CTX-M_, mcr-1* |
|  | 2 | Cecum | *bla_CTX-M_, mcr-1* |
|  | 3 | Cecum | *bla_CTX-M_, mcr-1, bla_TEM_* |
| **538** | 1 | Cecum | *bla_CTX-M_, mcr-1* |
| **539** | 1 | Cecum | *bla_CTX-M_, mcr-1, bla_TEM_* |
| **540** | 1 | Cecum | *bla_CTX-M_, mcr-1* |
|  | 2 | Cecum | *bla_CTX-M_, mcr-1* |
| **541** | 1 | Cecum | *bla_CTX-M_, mcr-1, bla_TEM_* |
| **542** | 1 | Cecum | *bla_CTX-M_, mcr-1* |
|  | 2 | Cecum | *bla_CTX-M_, mcr-1* |
|  | 3 | Cecum | *bla_CTX-M_, mcr-1* |
| **543** | 1 | Cecum | *bla_CTX-M_, mcr-1, bla_TEM_* |
| **545** | 1 | Cecum | *bla_CTX-M_, mcr-1, bla_TEM,_ bla_CMY_* |
|  | 2 | Cecum | *bla_CTX-M_, mcr-1, bla_TEM,_ bla_CMY_* |
| **604** | 1 | Cloacal swab on day 23 | *bla_CTX-M_, mcr-1, bla_CMY_* |
|  | 2 | Cloacal swab on day 23 | *bla_CTX-M,_  mcr-1, bla_CMY_* |

*****Animals 414 and 433 belonged to experimental group given CE via spray on day 1 of life, animals 502 to 545 belonged to experimental group given CE via drinking water on day 5 of life, and animal 604 was part of the positive control group. **^+^***mcr-1, bla_TEM_*, and *bla_CMY_* were plasmid-encoded from the donor pAmpC-producing *E. coli*; *bla_CTX-M_* was chromosomally encoded in the recipient ESBL-producing *E. coli* [33].

**S. Table 2.** Resistance genes detected under *in vitro conditions.*

|  | | | | **Resistance genes detected*** | | |
| --- | --- | --- | --- | --- | --- | --- |
| **Biological replicate** | **Initial bacterial concentration** | **Technical replicate** | **Isolate ID** | **LB** | **Intestinal chicken cells +DEMEM/F12** | **DEMEM/F12** |
| 1 | 10⁶ CFU/mL | 1 | 1 | *bla_CTX-M_, mcr-1* | *bla_CTX-M_, mcr-1* | *bla_CTX-M_, mcr-1* |
|  |  |  | 2 | *bla_CTX-M_, mcr-1* | *bla_CTX-M_, mcr-1* | *bla_CTX-M_, mcr-1, bla_TEM_* |
|  |  | 2 | 1 | *bla_CTX-M_, mcr-1* | *bla_CTX-M_, mcr-1* | *bla_CTX-M_, mcr-1* |
|  |  |  | 2 | *bla_CTX-M_, mcr-1* | *bla_CTX-M_, mcr-1, bla_TEM_* | *bla_CTX-M_, mcr-1* |
|  |  | 3 | 1 | *bla_CTX-M_, mcr-1* | *bla_CTX-M_, mcr-1* | *bla_CTX-M_, mcr-1, bla_TEM_* |
|  |  |  | 2 | *bla_CTX-M_, mcr-1* | *bla_CTX-M_, mcr-1, bla_TEM_* | *bla_CTX-M_, mcr-1* |
|  | 10⁵ CFU/mL | 1 | 1 | *bla_CTX-M_, mcr-1* | *bla_CTX-M_, mcr-1, bla_TEM_* | *bla_CTX-M_, mcr-1, bla_TEM_* |
|  |  |  | 2 | *bla_CTX-M_, mcr-1* | *bla_CTX-M_, mcr-1* | *bla_CTX-M_, mcr-1, bla_TEM_* |
|  |  | 2 | 1 | *bla_CTX-M_, mcr-1* | *bla_CTX-M_, mcr-1, bla_TEM_* | *bla_CTX-M_, mcr-1, bla_TEM_* |
|  |  |  | 2 | *bla_CTX-M_, mcr-1* | *bla_CTX-M_, mcr-1* | *bla_CTX-M_, mcr-1* |
|  |  | 3 | 1 | *bla_CTX-M_, mcr-1* | *bla_CTX-M_, mcr-1, bla_TEM_* | *bla_CTX-M_, mcr-1* |
|  |  |  | 2 | *bla_CTX-M_, mcr-1* | *bla_CTX-M_, mcr-1, bla_TEM_* | *bla_CTX-M_, mcr-1, bla_TEM_* |
| 2 | 10⁶ CFU/mL | 1 | 1 | *bla_CTX-M_, mcr-1* | *bla_CTX-M_, mcr-1* | *bla_CTX-M_, mcr-1* |
|  |  |  | 2 | *bla_CTX-M_, mcr-1* | *bla_CTX-M_, mcr-1* | *bla_CTX-M_, mcr-1* |
|  |  | 2 | 1 | *bla_CTX-M_, mcr-1* | *bla_CTX-M_, mcr-1* | *bla_CTX-M_, mcr-1* |
|  |  |  | 2 | *bla_CTX-M_, mcr-1* | *bla_CTX-M_, mcr-1* | *bla_CTX-M_, mcr-1, bla_TEM_* |
|  |  | 3 | 1 | *bla_CTX-M_, mcr-1* | *bla_CTX-M_, mcr-1* | *bla_CTX-M_, mcr-1* |
|  |  |  | 2 | *bla_CTX-M_, mcr-1* | *bla_CTX-M_, mcr-1* | *bla_CTX-M_, mcr-1* |
|  | 10⁵ CFU/mL | 1 | 1 | *bla_CTX-M_, mcr-1* | *bla_CTX-M_, mcr-1* | *bla_CTX-M_, mcr-1* |
|  |  |  | 2 | *bla_CTX-M_, mcr-1* | *bla_CTX-M_, mcr-1* | *bla_CTX-M_, mcr-1* |
|  |  | 2 | 1 | *bla_CTX-M_, mcr-1* | *bla_CTX-M_, mcr-1, bla_TEM,_ bla_CMY_* | *bla_CTX-M_, mcr-1, bla_TEM_* |
|  |  |  | 2 | *bla_CTX-M_, mcr-1* | *bla_CTX-M_, mcr-1, bla_TEM_* | *bla_CTX-M_, mcr-1, bla_TEM_* |
|  |  | 3 | 1 | *bla_CTX-M_, mcr-1* | *bla_CTX-M_, mcr-1* | *bla_CTX-M_, mcr-1, bla_TEM_* |
|  |  |  | 2 | *bla_CTX-M_, mcr-1* | *bla_CTX-M_, mcr-1* | *bla_CTX-M_, mcr-1, bla_TEM_* |
| 3 | 10⁶ CFU/mL | 1 | 1 | *bla_CTX-M_, mcr-1* | *bla_CTX-M_, mcr-1* | *bla_CTX-M_, mcr-1* |
|  |  |  | 2 | *bla_CTX-M_, mcr-1* | *bla_CTX-M_, mcr-1* | *bla_CTX-M_, mcr-1* |
|  |  | 2 | 1 | *bla_CTX-M_, mcr-1* | *bla_CTX-M_, mcr-1* | *bla_CTX-M_, mcr-1* |
|  |  |  | 2 | *bla_CTX-M_, mcr-1* | *bla_CTX-M_, mcr-1* | *bla_CTX-M_, mcr-1* |
|  |  | 3 | 1 | *bla_CTX-M_, mcr-1* | *bla_CTX-M_, mcr-1* | *bla_CTX-M_, mcr-1, bla_TEM_* |
|  |  |  | 2 | *bla_CTX-M_, mcr-1* | *bla_CTX-M_, mcr-1* | *bla_CTX-M_, mcr-1* |
|  | 10⁵ CFU/mL | 1 | 1 | *bla_CTX-M_, mcr-1* | *bla_CTX-M_, mcr-1, bla_TEM_, bla_CMY_* | *bla_CTX-M_, mcr-1* |
|  |  |  | 2 | *bla_CTX-M_, mcr-1* | *bla_CTX-M_, mcr-1* | *bla_CTX-M_, mcr-1* |
|  |  | 2 | 1 | *bla_CTX-M_, mcr-1* | *bla_CTX-M_, mcr-1* | *bla_CTX-M_, mcr-1* |
|  |  |  | 2 | *bla_CTX-M_, mcr-1* | *bla_CTX-M_, mcr-1* | *bla_CTX-M_, mcr-1* |
|  |  | 3 | 1 | *bla_CTX-M_, mcr-1* | *bla_CTX-M_, mcr-1* | *bla_CTX-M_, mcr-1, bla_TEM_, bla_CMY_* |
|  |  |  | 2 | *bla_CTX-M_, mcr-1* | *bla_CTX-M_, mcr-1* | *bla_CTX-M_, mcr-1* |
| 4 | 10⁶ CFU/mL | 1 | 1 | *bla_CTX-M_, mcr-1* | *bla_CTX-M_, mcr-1* | *bla_CTX-M_, mcr-1* |
|  |  |  | 2 | *bla_CTX-M_, mcr-1* | *bla_CTX-M_, mcr-1* | *bla_CTX-M_, mcr-1* |
|  |  | 2 | 1 | *bla_CTX-M_, mcr-1* | *bla_CTX-M_, mcr-1* | *bla_CTX-M_, mcr-1* |
|  |  |  | 2 | *bla_CTX-M_, mcr-1* | *bla_CTX-M_, mcr-1* | *bla_CTX-M_, mcr-1* |
|  |  | 3 | 1 | *bla_CTX-M_, mcr-1* | *bla_CTX-M_, mcr-1* | *bla_CTX-M_, mcr-1* |
|  |  |  | 2 | *bla_CTX-M_, mcr-1* | *bla_CTX-M_, mcr-1, bla_TEM_* | *bla_CTX-M_, mcr-1* |
|  | 10⁵ CFU/mL | 1 | 1 | *bla_CTX-M_, mcr-1* | *bla_CTX-M_, mcr-1* | *bla_CTX-M_, mcr-1, bla_TEM_* |
|  |  |  | 2 | *bla_CTX-M_, mcr-1* | *bla_CTX-M_, mcr-1* | *bla_CTX-M_, mcr-1, bla_TEM_, bla_CMY_* |
|  |  | 2 | 1 | *bla_CTX-M_, mcr-1* | *bla_CTX-M_, mcr-1* | *bla_CTX-M_, mcr-1* |
|  |  |  | 2 | *bla_CTX-M_, mcr-1* | *bla_CTX-M_, mcr-1* | *bla_CTX-M_, mcr-1, bla_TEM_* |
|  |  | 3 | 1 | *bla_CTX-M_, mcr-1* | *bla_CTX-M_, mcr-1* | *bla_CTX-M_, mcr-1, bla_TEM_* |
|  |  |  | 2 | *bla_CTX-M_, mcr-1* | *bla_CTX-M_, mcr-1* | *bla_CTX-M_, mcr-1* |
| 5 | 10⁶ CFU/mL | 1 | 1 | *bla_CTX-M_, mcr-1* | *bla_CTX-M_, mcr-1* | *bla_CTX-M_, mcr-1* |
|  |  |  | 2 | *bla_CTX-M_, mcr-1* | *bla_CTX-M_, mcr-1* | *bla_CTX-M_, mcr-1* |
|  |  | 2 | 1 | *bla_CTX-M_, mcr-1* | *bla_CTX-M_, mcr-1* | *bla_CTX-M_, mcr-1, bla_TEM_* |
|  |  |  | 2 | *bla_CTX-M_, mcr-1* | *bla_CTX-M_, mcr-1* | *bla_CTX-M_, mcr-1* |
|  |  | 3 | 1 | *bla_CTX-M_, mcr-1* | *bla_CTX-M_, mcr-1* | *bla_CTX-M_, mcr-1* |
|  |  |  | 2 | *bla_CTX-M_, mcr-1* | *bla_CTX-M_, mcr-1* | *bla_CTX-M_, mcr-1* |
|  | 10⁵ CFU/mL | 1 | 1 | *bla_CTX-M_, mcr-1* | *bla_CTX-M_, mcr-1* | *bla_CTX-M_, mcr-1, bla_TEM_* |
|  |  |  | 2 | *bla_CTX-M_, mcr-1* | *bla_CTX-M_, mcr-1* | *bla_CTX-M_, mcr-1* |
|  |  | 2 | 1 | *bla_CTX-M_, mcr-1* | *bla_CTX-M_, mcr-1* | *bla_CTX-M_, mcr-1, bla_TEM_, bla_CMY_* |
|  |  |  | 2 | *bla_CTX-M_, mcr-1* | *bla_CTX-M_, mcr-1* | *bla_CTX-M_, mcr-1* |
|  |  | 3 | 1 | *bla_CTX-M_, mcr-1* | *bla_CTX-M_, mcr-1* | *bla_CTX-M_, mcr-1, bla_TEM_, bla_CMY_* |
|  |  |  | 2 | *bla_CTX-M_, mcr-1* | *bla_CTX-M_, mcr-1* | *bla_CTX-M_, mcr-1* |
| 6 | 10⁶ CFU/mL | 1 | 1 | *bla_CTX-M_, mcr-1* | *bla_CTX-M_, mcr-1* | *bla_CTX-M_, mcr-1* |
|  |  |  | 2 | *bla_CTX-M_, mcr-1* | *bla_CTX-M_, mcr-1* | *bla_CTX-M_, mcr-1, bla_TEM_, bla_CMY_* |
|  |  | 2 | 1 | *bla_CTX-M_, mcr-1* | *bla_CTX-M_, mcr-1* | *bla_CTX-M_, mcr-1* |
|  |  |  | 2 | *bla_CTX-M_, mcr-1* | *bla_CTX-M_, mcr-1, bla_TEM_* | *bla_CTX-M_, mcr-1* |
|  |  | 3 | 1 | *bla_CTX-M_, mcr-1* | *bla_CTX-M_, mcr-1* | *bla_CTX-M_, mcr-1* |
|  |  |  | 2 | *bla_CTX-M_, mcr-1* | *bla_CTX-M_, mcr-1* | *bla_CTX-M_, mcr-1* |
|  | 10⁵ CFU/mL | 1 | 1 | *bla_CTX-M_, mcr-1* | *bla_CTX-M_, mcr-1, bla_TEM_* | *bla_CTX-M_, mcr-1* |
|  |  |  | 2 | *bla_CTX-M_, mcr-1* | *bla_CTX-M_, mcr-1, bla_TEM_* | *bla_CTX-M_, mcr-1* |
|  |  | 2 | 1 | *bla_CTX-M_, mcr-1* | *bla_CTX-M_, mcr-1, bla_TEM_, bla_CMY_* | *bla_CTX-M_, mcr-1* |
|  |  |  | 2 | *bla_CTX-M_, mcr-1* | *bla_CTX-M_, mcr-1* | *bla_CTX-M_, mcr-1* |
|  |  | 3 | 1 | *bla_CTX-M_, mcr-1* | *bla_CTX-M_, mcr-1, bla_TEM_, bla_CMY_* | *bla_CTX-M_, mcr-1* |
|  |  |  | 2 | *bla_CTX-M_, mcr-1* | *bla_CTX-M_, mcr-1, bla_TEM_* | *bla_CTX-M_, mcr-1* |
| 7 | 10⁶ CFU/mL | 1 | 1 | *bla_CTX-M_, mcr-1* | *bla_CTX-M_, mcr-1, bla_TEM_* | *bla_CTX-M_, mcr-1* |
|  |  |  | 2 | *bla_CTX-M_, mcr-1* | *bla_CTX-M_, mcr-1* | *bla_CTX-M_, mcr-1, bla_TEM_* |
|  |  | 2 | 1 | *bla_CTX-M_, mcr-1* | *bla_CTX-M_, mcr-1* | *bla_CTX-M_, mcr-1* |
|  |  |  | 2 | *bla_CTX-M_, mcr-1* | *bla_CTX-M_, mcr-1* | *bla_CTX-M_, mcr-1, bla_TEM_* |
|  |  | 3 | 1 | *bla_CTX-M_, mcr-1* | *bla_CTX-M_, mcr-1* | *bla_CTX-M_, mcr-1* |
|  |  |  | 2 | *bla_CTX-M_, mcr-1* | *bla_CTX-M_, mcr-1* | *bla_CTX-M_, mcr-1* |
|  | 10⁵ CFU/mL | 1 | 1 | *bla_CTX-M_, mcr-1* | *bla_CTX-M_, mcr-1, bla_TEM_* | *bla_CTX-M_, mcr-1* |
|  |  |  | 2 | *bla_CTX-M_, mcr-1* | *bla_CTX-M_, mcr-1* | *bla_CTX-M_, mcr-1, bla_TEM_, bla_CMY_* |
|  |  | 2 | 1 | *bla_CTX-M_, mcr-1* | *bla_CTX-M_, mcr-1, bla_TEM_* | *bla_CTX-M_, mcr-1* |
|  |  |  | 2 | *bla_CTX-M_, mcr-1* | *bla_CTX-M_, mcr-1* | *bla_CTX-M_, mcr-1* |
|  |  | 3 | 1 | *bla_CTX-M_, mcr-1* | *bla_CTX-M_, mcr-1, bla_TEM_, bla_CMY_* | *bla_CTX-M_, mcr-1* |
|  |  |  | 2 | *bla_CTX-M_, mcr-1* | *bla_CTX-M_, mcr-1* | *bla_CTX-M_, mcr-1* |

***** *mcr-1, bla_TEM_*, and *bla_CMY_* were plasmid-encoded from the donor pAmpC-producing *E. coli*; *bla_CTX-M_* was chromosomally encoded in the recipient ESBL-producing *E. coli* [33].

**S. Table 3.** Cecal conjugation frequencies in broiler chickens after 49-day fattening period.

| **Animal number*** | **Transconjugants**  **(log_10_CFU/mL)** | **ESBL-Recipient**  **(log_10_CFU/mL)** | **pAmpC-Donor**  **(log_10_CFU/mL)** | **Transconjugants relative to donor (log_10_CFU/mL)** |
| --- | --- | --- | --- | --- |
| **414** | 1.60 | 6.72 | 6.72 | -5.12 |
| **433** | 1.60 | 6.15 | 6.41 | -4.81 |
| **502** | 1.30 | 6.33 | 6.00 | -4.70 |
| **503** | 1.30 | 6.63 | 6.51 | -5.21 |
| **505** | 1.78 | 6.96 | 6.82 | -5.04 |
| **506** | 1.30 | 6.91 | 6.35 | -5.05 |
| **507** | 1.90 | 6.75 | 6.59 | -4.69 |
| **508** | 1.30 | 6.35 | 5.79 | -4.49 |
| **509** | 1.90 | 5.58 | 6.49 | -4.59 |
| **511** | 1.30 | 7.97 | 6.99 | -5.69 |
| **512** | 1.78 | 6.79 | 5.20 | -3.43 |
| **513** | 1.30 | 7.26 | 5.78 | -4.48 |
| **516** | 2.00 | 8.85 | 7.14 | -5.14 |
| **517** | 1.60 | 6.61 | 5.64 | -4.04 |
| **519** | 1.30 | 6.70 | 6.55 | -5.25 |
| **521** | 1.30 | 6.11 | 6.15 | -4.85 |
| **522** | 1.30 | 6.64 | 6.45 | -5.15 |
| **523** | 1.30 | 6.49 | 6.76 | -5.46 |
| **524** | 1.30 | 6.59 | 6.55 | -5.25 |
| **525** | 1.90 | 6.94 | 6.84 | -4.93 |
| **526** | 1.60 | 6.95 | 7.04 | -5.44 |
| **527** | 1.30 | 7.06 | 6.82 | -5.51 |
| **528** | 1.30 | 6.95 | 6.42 | -5.12 |
| **529** | 1.90 | 6.98 | 6.64 | -4.74 |
| **530** | 1.30 | 7.04 | 6.90 | -5.60 |
| **531** | 1.78 | 6.77 | 6.31 | -4.54 |
| **534** | 1.60 | 8.44 | 6.40 | -4.80 |
| **536** | 1.78 | 6.75 | 6.01 | -4.23 |
| **538** | 1.30 | 7.79 | 7.72 | -6.41 |
| **539** | 1.30 | 7.06 | 5.81 | -4.51 |
| **540** | 1.60 | 7.08 | 6.91 | -5.31 |
| **541** | 1.30 | 8.43 | 6.39 | -5.09 |
| **542** | 1.78 | 8.72 | 6.36 | -4.58 |
| **543** | 1.30 | 6.72 | 6.61 | -5.31 |
| **545** | 1.60 | 6.66 | 8.60 | -7.00 |

Frequencies expressed as transconjugants relative donor (log_10_CFU/mL) resulting in the transfer of *mcr-1* gene from donor pAmpC-producing *E. coli* to recipient ESBL-producing *E. coli*. *Animals 414 and 433 belonged to experimental group given CE via spray on day 1 of life. Animals 502 to 545 belonged to experimental group given CE via drinking water on day 5 of life.

**S. Table 4.** Conjugation frequencies under in vitro conditions.

| **Biological replicate** | **Transconjugants relative to donor**  **(log_10_CFU/mL)** | | | | | |
| --- | --- | --- | --- | --- | --- | --- |
|  | **LB Broth** | | **Intestinal chicken cells** | | **DMEM/F-12** | |
|  | **10⁵ CFU/mL** | **10⁶ CFU/mL** | **10⁵ CFU/mL** | **10⁶ CFU/mL** | **10⁵ CFU/mL** | **10⁶ CFU/mL** |
| **1** | -5.94 | -5.80 | -6.82 | -6.96 | -7.12 | -6.30 |
|  | -6.06 | -6.15 | -6.48 | -7.12 | -6.93 | -7.08 |
|  | -6.20 | -5.85 | -6.91 | -7.22 | -7.35 | -6.72 |
| **2** | -6.33 | -6.93 | -6.40 | -6.90 | -6.70 | -7.22 |
|  | -6.66 | -6.48 | -6.24 | -6.54 | -6.91 | -7.09 |
|  | -6.59 | -6.42 | -6.38 | -7.28 | -6.45 | -6.77 |
| **3** | -6.32 | -6.30 | -6.81 | -6.89 | -7.67 | -7.52 |
|  | -6.46 | -6.69 | -6.84 | -7.57 | -6.85 | -7.64 |
|  | -6.53 | -6.81 | -6.94 | -7.89 | -6.92 | -7.32 |
| **4** | -6.16 | -6.33 | -7.41 | -6.76 | -7.40 | -6.62 |
|  | -6.11 | -6.24 | -6.82 | -6.70 | -7.40 | -6.95 |
|  | -6.03 | -6.68 | -6.49 | -6.97 | -7.38 | -6.50 |
| **5** | -6.30 | -6.44 | -6.53 | -6.28 | -6.11 | -6.58 |
|  | -6.11 | -6.30 | -6.28 | -6.31 | -6.07 | -6.85 |
|  | -6.10 | -6.51 | -6.10 | -6.33 | -6.15 | -6.22 |
| **6** | -5.76 | -6.75 | -6.53 | -6.60 | -6.57 | -6.74 |
|  | -6.28 | -5.95 | -6.49 | -6.67 | -6.56 | -6.82 |
|  | -6.03 | -5.97 | -6.52 | -6.78 | -6.64 | -6.81 |
| **7** | -6.44 | -6.69 | -6.75 | -7.07 | -6.79 | -6.38 |
|  | -6.29 | -6.59 | -6.73 | -6.75 | -6.86 | -6.85 |
|  | -6.56 | -6.65 | -6.46 | -7.36 | -6.75 | -6.46 |

Frequencies expressed as transconjugants relative donor (log_10_CFU/mL) resulting in the transfer of *mcr-1* gene from donor pAmpC-producing *E. coli* to recipient ESBL-producing *E. coli*, under three different in vitro conditions: LB broth, intestinal chicken cells, and DMEM/F-12 cell culture medium, using two initial bacterial concentrations (10⁵ and 10⁶ CFU/mL). Data represents seven biological replicates, each with three technical replicates.

**S. Table 5.** Global one-way Anova.

| **Effect** | **Estimate** | **95% Confidence Interval** | **p-value** |
| --- | --- | --- | --- |
| one-way ANOVA | η² = 0.409 | 0.089 – 0.532 | 0.001 |

| **Group** | **Group** | **Mean Difference** | **p-value** | **95% CI Lower** | **95% CI Upper** |
| --- | --- | --- | --- | --- | --- |
| Intestinal cells in DMEM/F-12 (10^5^CFU/mL) | Intestinal cells in DMEM/F-12 (10^6^CFU/mL) | 0.26 | 0.80 | -0.24 | 0.77 |
| Intestinal cells in DMEM/F-12 (10^5^CFU/mL) | DMEM/F-12 (10^5^CFU/mL) | 0.23 | 0.91 | -0.28 | 0.74 |
| Intestinal cells in DMEM/F-12 (10^5^CFU/mL) | LB 10^5^CFU/mL | -0.35 | 0.42 | -0.86 | 0.16 |
| Intestinal cells in DMEM/F-12 (10^6^CFU/mL) | DMEM/F-12 (10^6^CFU/mL) | -0.06 | 1.00 | -0.57 | 0.44 |
| Intestinal cells in DMEM/F-12 (10^6^CFU/mL) | LB 10^6^CFU/mL | -0.48 | 0.07 | -0.99 | 0.02 |
| DMEM/F-12 (10^5^CFU/mL) | DMEM/F-12 (10^6^CFU/mL) | -0.03 | 1.00 | -0.53 | 0.48 |
| DMEM/F-12 (10^5^CFU/mL) | LB 10^5^CFU/mL | -0.58 | 0.02 | -1.08 | -0.07 |
| DMEM/F-12 (10^6^CFU/mL) | LB 10^6^CFU/mL | -0.42 | 0.18 | -0.93 | 0.09 |
| LB 10^5^CFU/mL | LB 10^6^CFU/mL | 0.13 | 1.00 | -0.38 | 0.64 |

**S. Table 6.** Hochberg-adjusted post hoc comparisons.
